# Detection and Monitoring of Interstitial Lung Disease in Patients with Systemic Sclerosis

**DOI:** 10.1007/s11926-022-01067-5

**Published:** 2022-05-01

**Authors:** Surabhi Agarwal Khanna, John W. Nance, Sally A. Suliman

**Affiliations:** 1 University of Cincinnati, Cincinnati, OH USA; 2 Houston Methodist Hospital, Houston, TX USA; 3 University of Arizona, Phoenix, AZ USA

**Keywords:** Autoimmune diseases, Connective tissue disease, Pulmonary fibrosis, Scleroderma

## Abstract

**Purpose of Review:**

Interstitial lung disease (ILD) is a common manifestation of systemic sclerosis (SSc). We explore the importance of early detection, monitoring, and management of SSc-ILD.

**Recent Findings:**

All patients with SSc are at risk of ILD and should be screened for ILD at diagnosis using a high-resolution computed tomography (HRCT) scan. Some patients with SSc-ILD develop a progressive phenotype characterized by worsening fibrosis on HRCT, decline in lung function, and early mortality. To evaluate progression and inform treatment decisions, regular monitoring is important and should include pulmonary function testing, evaluation of symptoms and quality of life, and, where indicated, repeat HRCT. Multidisciplinary discussion enables comprehensive evaluation of the available information and its implications for management. The first-line treatment for SSc-ILD is usually immunosuppression. The antifibrotic drug nintedanib has been approved for slowing lung function decline in patients with SSc-ILD.

**Summary:**

Optimal management of patients with SSc-ILD requires a multidisciplinary and patient-centered approach.

## Introduction

Systemic sclerosis (SSc) is a rare multifaceted autoimmune disease characterized by progressive fibrosis of the skin and internal organs, immune dysregulation, and vasculopathy [1]. Interstitial lung disease (ILD) is a common manifestation of SSc and contributes significantly to the overall morbidity and mortality of patients with SSc. The pathogenesis of SSc-ILD involves multiple components including interstitial inflammation and fibrosis [2]. In some patients, SSc-ILD is progressive, with poor outcomes including premature death [3]. In this article, we explore the importance of early detection, monitoring, and management of ILD in patients with SSc.

## Detection of SSc-ILD

**Fig. 1:**
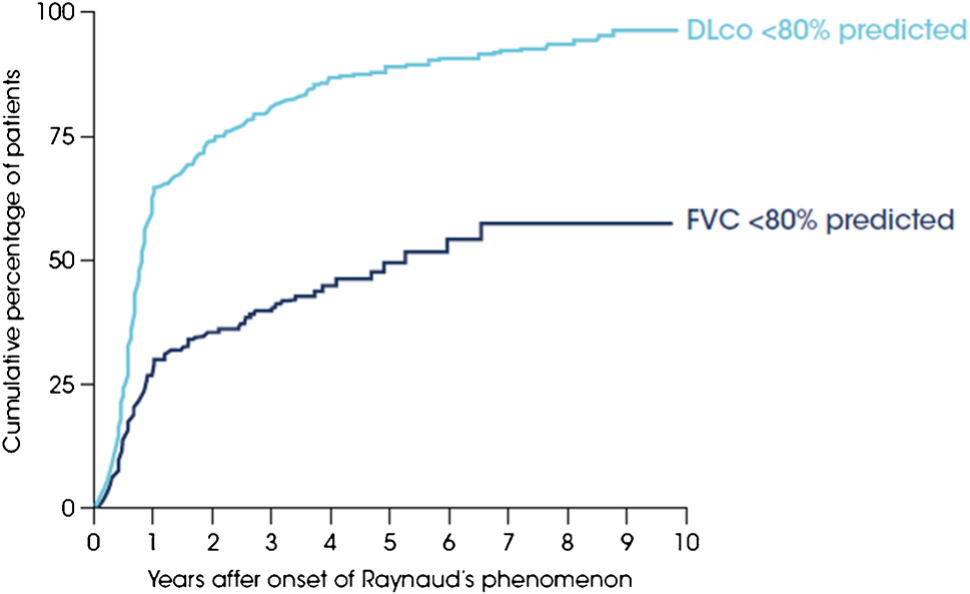
Early development of lung function impairment in patients with SSc in the EUSTAR database (adapted with permission from [7])

SSc-ILD is classified as pulmonary fibrosis seen on high-resolution computed tomography (HRCT) or chest radiography, with a basilar predominance, or the occurrence of “Velcro” crackles on chest auscultation, not due to another cause such as congestive heart failure [1]. The estimated prevalence of ILD in patients with SSc varies widely depending on the population studied and the methodology used. In a Canadian registry, 64% of 289 patients with SSc who had HRCT scans were diagnosed with ILD, compared with 22% of 1168 patients who had a chest X-ray [4]. In a Norwegian population-based cohort of 650 patients with SSc, ILD was evident on HRCT in half the patients [3]. Risk factors for the development of ILD in patients with SSc include male sex, diffuse cutaneous SSc (dcSSc), African-American race, and the presence of certain autoantibodies such as anti-topoisomerase I (ATA), anti-Th/To, and anti-U3 RNP [5, 6]. The risk of developing ILD is greatest in the first few years after diagnosis of SSc [7] (Fig. 1). However, it is important to be aware that patients with SSc with no established risk factors are still at risk of developing ILD. A recent analysis of data from 826 patients with SSc-ILD in the EUSTAR database found that only 21% of patients had a disease duration < 3 years, 50% had dcSSc, and 53% were ATA-positive [8••]. This highlights the importance of not assuming that only patients with early dcSSc are at risk of SSc-ILD.

While a chronic dry cough and dyspnea are common symptoms in patients with SSc-ILD, many patients remain asymptomatic [9]. Furthermore, patients with SSc with evidence of ILD on HRCT may have normal pulmonary function tests (PFTs) [10, 11]. In a retrospective analysis of 188 patients with SSc-ILD on HRCT, 31% of patients had FVC ≥ 80% predicted [11]. Thus relying on symptoms, or impaired lung function to drive screening for SSc-ILD would result in missed diagnoses. In Delphi consensus exercises, expert groups in the USA and in Europe agreed that all patients diagnosed with SSc should undergo a HRCT scan of the chest at baseline to screen for ILD [12, 13••].

**Fig. 2:**
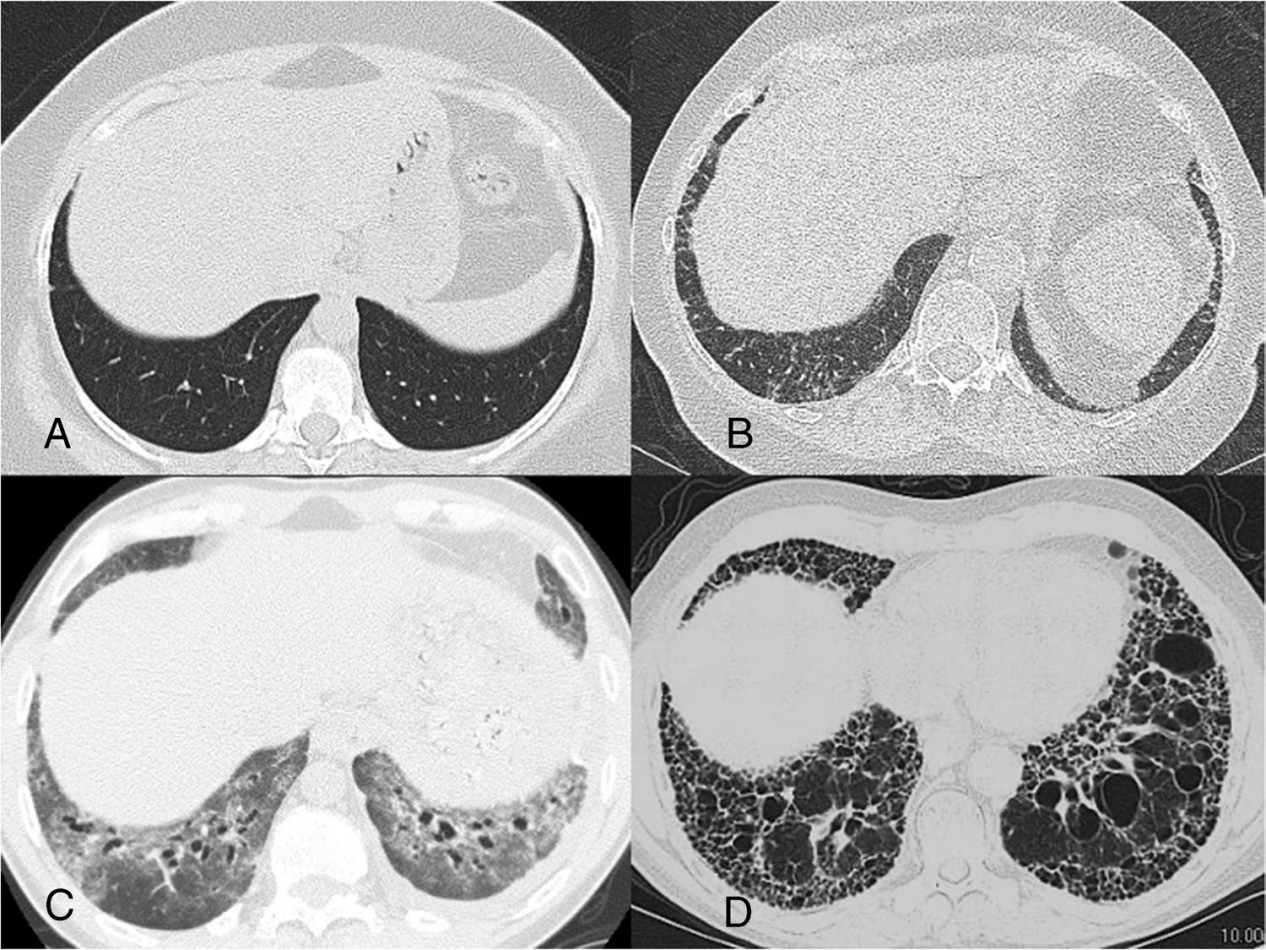
Examples of imaging patterns seen in patients with SSc. **a** Axial HRCT image from the normal lung bases of a 55-year-old woman undergoing staging for pancreatic cancer. The only visible structures are bronchovascular bundles and pleural reflections; there are no interstitial lung abnormalities. **b** Early fibrotic ILD in a 47-year-old woman with SSc. There are many crossing lines (reticulations), indicating early fibrotic lung disease. It is difficult to apply a specific pattern at this early stage. **c** An NSIP pattern in a 52-year-old woman with SSc. The pattern is characterized by ground-glass opacities, traction bronchiectasis, and subpleural sparing. **d** A UIP pattern of fibrotic ILD in a 59-year-old man with SSc. There are extensive reticulations and honeycombing throughout the basilar and peripheral lungs

HRCT scans from a patient without interstitial lung abnormalities and a patient with early fibrotic lung disease are shown in Figs. 2a and b. In addition to identifying the presence of ILD, HRCT enables evaluation of the extent and pattern of fibrosis. This is important, as a greater extent of fibrotic ILD on HRCT is associated with poorer outcomes [3, 14, 15], as are specific imaging features, such as traction bronchiectasis and honeycombing [16]. Establishing an imaging pattern in connective tissue disease-related ILDs is difficult and subject to high inter-observer variability [17, 18]. The most common imaging pattern observed on HRCT in patients with SSc-ILD is non-specific interstitial pneumonia (NSIP), characterized by bilateral ground-glass opacities, reticulation and traction bronchiectasis that is most prominent in the lower lobes (Fig. 2c); however, some patients with SSc-ILD show a usual interstitial pneumonia (UIP) pattern (Fig. 2d) [19]. In addition, a number of patients do not fulfill the definition for any specific pattern. It is important to note that the imaging classification paradigm is continuously evolving, and it is more important to recognize the presence of new or evolving ILD than the specific fibrotic pattern. Table 1 reviews the standard and non-standard lexicons used in the setting of fibrosis in thoracic radiology. Although some terms are more specific to fibrotic ILD than others, given the heterogeneity in experience and reporting standards among radiologists, identification of any of these words should prompt the referring clinician to question the presence of ILD.

**Table 1:** Lexicons in thoracic radiology in the setting of fibrosis

Level of descriptor	Term	Description
Specific imaging features (adapted from [51])	Architectural distortion	Abnormal displacement of bronchi, vessels, or fissures
	Ground-glass opacity	Lung attenuation that is abnormally increased but does not obscure underlying bronchovascular structures
	Honeycombing	Stacked subpleural cystic spaces indicative of end-stage parenchymal fibrosis
	Interstitial opacities/lines/thickening/fibrosis	Thickening of the connective tissues of the lung, resulting in too many and too prominent lines. Sometimes used interchangeably with “reticulations”
	Parenchymal band	A thicker (1–3 mm) linear opacity, usually extending to the visceral pleura
	Reticulation	Innumerable small linear opacities
	Traction bronchiectasis/bronchiolectasis	Irregular bronchial/bronchiolar dilatation due to cicatrizing surrounding fibrosis
Patterns	Usual interstitial pneumonia	Basilar, subpleural predominant fibrotic ILD with a preponderance of reticulations and honeycombing when more severe
	Non-specific interstitial pneumonia	Usually basilar, subpleural predominant fibrotic ILD that is characterized more by ground-glass opacities and traction bronchiectasis/bronchiolectasis than reticulations and honeycombing
	Organizing pneumonia	There is a wide spectrum of imaging findings, but it most commonly presents as multifocal peribronchovascular and subpleural consolidations
Other phrases that should prompt attention	Fibrotic ILD	These phrases are commonly used when the interpreting physician does not want to specify a pattern or diagnosis
	Non-specific or unclassifiable fibrosis	
	Fibrotic-like changes	
	Confluent basilar scarring	
	Subpleural cysts	Often used in place of “honeycombing” when there is doubt as to the presence of true honeycombing

## Progression and Monitoring of SSc-ILD

SSc-ILD has a variable natural history [20]. Some patients have relatively stable disease for a prolonged period, while others develop a progressive phenotype characterized by worsening fibrosis on HRCT, progressive decline in lung function, worsening dyspnea, and an increase in mortality [3]. Risk factors for the progression of SSc-ILD include advanced age, low baseline FVC and/or DLco, a greater extent of ILD on HRCT, the presence of ATA, and the absence of anti-centromere antibodies [6, 14, 15, 21, 22].

**Fig. 3:**
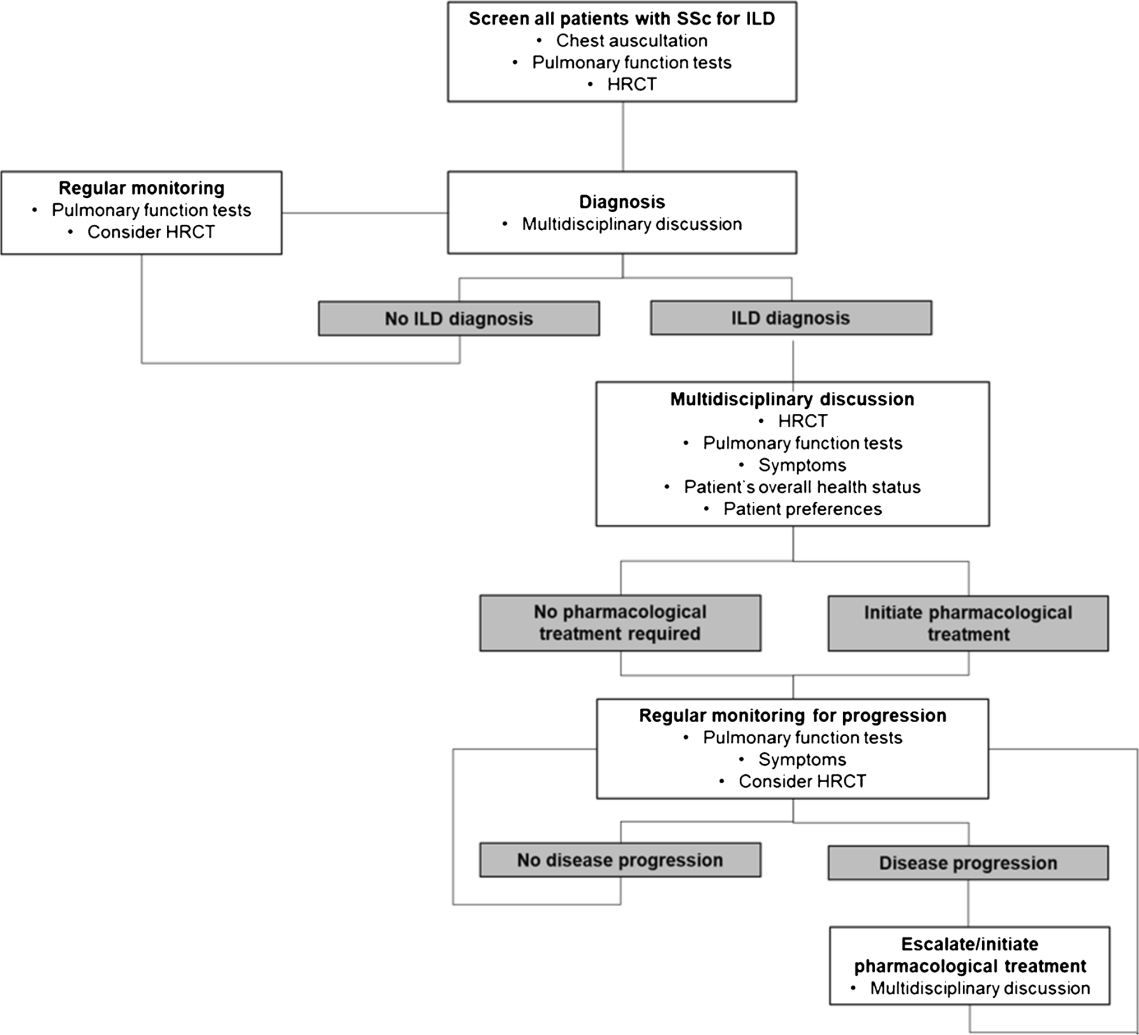
Proposed algorithm for the detection, monitoring, and management of patients with SSc-ILD (reproduced with permission from [13••])

**Fig. 4:**
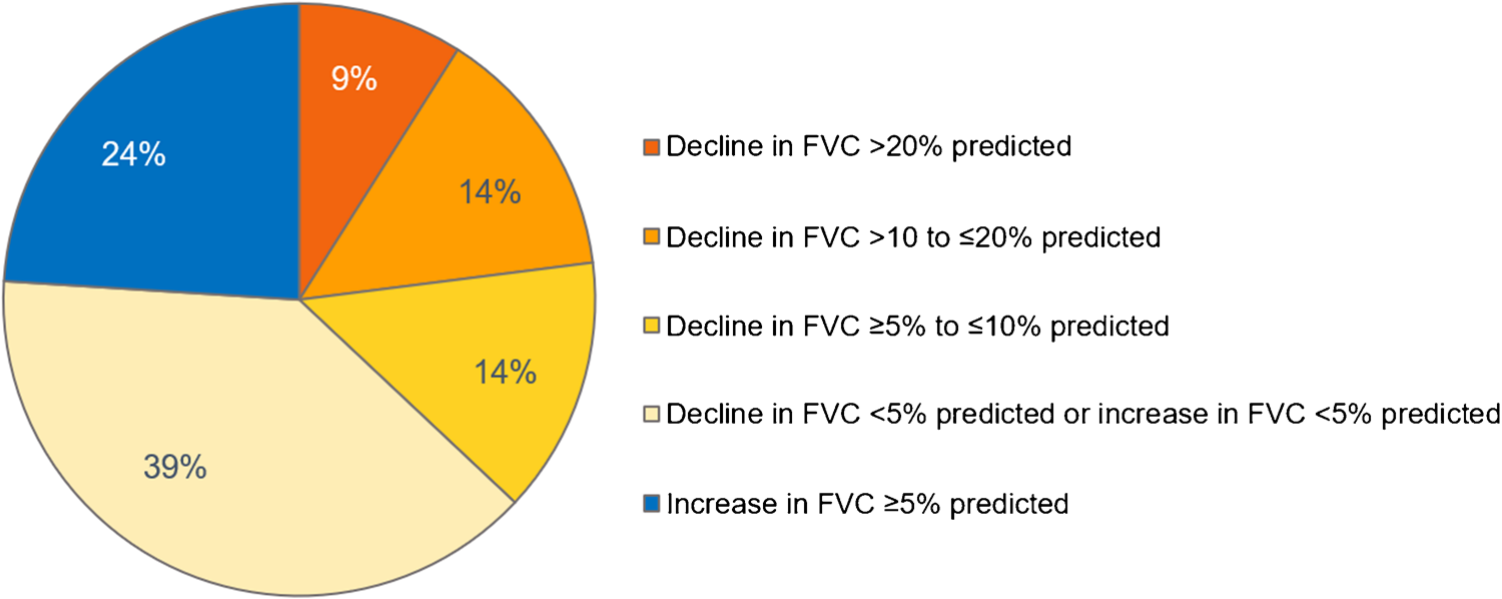
Change in FVC over a mean follow-up of 5 years in patients with SSc-ILD in the EUSTAR database [8••]

Patients with SSc-ILD should undergo regular PFTs to evaluate lung function and monitor disease severity and rapidity of progression (Fig. 3) [13••]. In an analysis of 535 patients with SSc-ILD in the EUSTAR database, over a mean follow-up of 5 years, 63% had stable or improved FVC (defined as a decline in FVC < 5% predicted or an increase in FVC) while 23% had a decline in FVC of > 10% predicted (Fig. 4) [8••]. It is important to note that recent stability in FVC does not indicate that FVC will remain stable over the following year [8••]. Among patients with SSc-ILD in the EUSTAR database who had stable FVC (defined as a decline or improvement of < 5% predicted) over a 12-month period, approximately 30% had a decline in FVC of ≥ 5% predicted over the following 12 months [8••].

As well as PFTs, monitoring of patients with SSc-ILD should include evaluation of symptoms, quality of life, exercise-induced oxygen desaturation, and repeat HRCT where indicated (e.g., if there has been a worsening of PFTs or symptoms) (Fig. 3) [13••]. Protocols for monitoring patients with SSc-ILD vary, but most experts agree that PFTs and respiratory symptoms should be assessed at least every 6–12 months and screening for pulmonary hypertension, a common comorbidity of SSc-ILD [23], should be performed annually via echocardiogram. Rheumatologists are advised to refer patients with SSc-ILD to an expert ILD center, or to establish a relationship with such a center. These centers can monitor disease progression closely and adopt a multidisciplinary approach to ensure re-evaluation of a patient’s therapeutic regimen.

While there is no well-established definition for progression of SSc-ILD, experts have proposed that a decline in FVC of ≥ 10% of the predicted value, or a decline in FVC of 5–9% of the predicted value accompanied by a decline in DLco of ≥ 15% of the predicted value, may represent clinically meaningful progression [24]. When interpreting the results of PFTs, it is important to consider whether other factors may be contributing to an observed decline, e.g., a worsening of pulmonary hypertension or a concomitant respiratory infection. The same applies to an observed worsening in dyspnea, cough, or quality of life: a thorough investigation is needed to ascertain whether such a worsening is due to progression of ILD rather than to the onset or worsening of other manifestations of SSc or comorbidities. “Small” deteriorations in PFTs should not be ignored as these may accumulate over time and are associated with an increased risk of mortality [3, 22, 25]. Among 228 patients with SSc-ILD in a Norwegian cohort, after patients were stratified by change in FVC over a mean follow-up of 6.2 years, the 10-year survival rate was 59% in patients with a decline in FVC of ≥ 5% predicted compared to 78% in patients with a decline or an increase < 5% predicted [3].

Furthermore, an increase in the extent of fibrotic abnormalities on HRCT indicates disease progression. A seminal study at a UK center found that extensive ILD, defined as > 30% extent of fibrosis on HRCT (based on a mean score of five sections, each estimated to the nearest 5%) or an extent of fibrosis on HRCT of 10–30% with an FVC < 70% predicted, was strongly predictive of mortality (hazard ratio 3.46) [14]. While most studies have focused on analyses of thresholds, any increase in the extent of fibrotic abnormalities on HRCT has prognostic implications [3, 26]. Automated post-processing software is available to quantify the extent of fibrotic changes on CT [27] but is rarely used in a routine clinical environment. Data are mixed on the prognostic value of specific imaging patterns in patients with SSc-ILD, with some studies showing no prognostic value of a UIP pattern compared with NSIP [28] and others showing worse outcomes in patients with a UIP pattern on HRCT [29].

Multidisciplinary discussion involving, at minimum, a rheumatologist, pulmonologist, and radiologist enables a comprehensive evaluation of the information obtained during monitoring and its implications for the care of the patient. Multidisciplinary discussion may take place virtually or at face-to-face meetings. Input from all parties is valuable: the rheumatologist can provide the most complete picture of the patient’s overall health status, the pulmonologist can guide the interpretation of PFTs, and the radiologist can advise on changes observed on HRCT that may have prognostic relevance.

## Management of SSc-ILD

There is no established algorithm for when patients with SSc-ILD should receive pharmacological therapy or which treatments they should receive. However, there is an increasing recognition of the importance of early treatment of SSc-ILD to improve patient outcomes, given that lung function lost to fibrosis cannot be recovered and there is a strong association between decline in lung function and mortality. The information gathered through monitoring should be discussed from the perspective of whether a change or escalation of therapy is required. Decisions about treatment should be based on assessment of the patient’s overall health status, ascertainment of ILD severity and progression, risk factors for ILD progression, and consideration of the patient’s preferences [13••, 30].

In most patients, the first-line treatment for SSc-ILD is immunosuppression, and many patients with SSc-ILD will in any case be taking an immunomodulatory therapy to manage other manifestations of their SSc, such as skin fibrosis [31]. The most commonly used immunosuppressant therapy in patients with SSc-ILD is mycophenolate, which is better tolerated than cyclophosphamide and non-inferior to cyclophosphamide in its effects on lung function [32]. In Scleroderma Lung Study I (SLS I), performed in 158 patients with SSc-ILD, the mean decline in FVC % predicted after 1 year was 1.0% in patients treated with oral cyclophosphamide compared with 2.6% in the placebo group [33]. Later, in SLS II, conducted in 142 patients, mean improvements in FVC % predicted were similar between patients treated with mycophenolate for 2 years and patients treated with oral cyclophosphamide for 1 year followed by placebo for 1 year (2.2% and 2.9%, respectively) [32]. Mycophenolate is associated with a risk of gastrointestinal intolerance and infections but can be administered for prolonged periods. The side effects of cyclophosphamide limit its long-term use [34].

Tocilizumab, an interleukin-6 (IL-6) receptor antagonist, was investigated as a treatment for SSc in two placebo-controlled trials in patients with early inflammatory dcSSc: the phase II faSScinate trial [35] and the phase III focuSSced trial [36]. The primary endpoint, change in modified Rodnan skin score (mRSS), was not met in either of these trials, but secondary endpoints suggested a beneficial effect of tocilizumab on FVC. In the focuSSced trial, among 210 patients with SSc, the mean change from baseline in FVC % predicted at week 48 was − 0.4% in the tocilizumab group compared with − 4.6% in the placebo group [36]. In the subgroup of 136 patients with SSc-ILD at baseline (defined as ground-glass opacification and/or fibrosis with a basal predominance on HRCT), the mean change from baseline in FVC % predicted at week 48 was − 0.1% in patients treated with tocilizumab compared with − 6.3% in the placebo group [37••]. Based on these findings, tocilizumab has been approved by the US Food and Drug Administration for slowing the decline in lung function in patients with SSc-ILD. Adverse events associated with tocilizumab include infections, liver enzyme elevations, and injection site reactions [38].

Open-label and observational studies suggest that rituximab, a monoclonal anti-CD20 antibody that depletes B-cells, may have a beneficial effect on lung function in patient with SSc-ILD [39], but evidence from randomized, double-blind, controlled trials is lacking. The results of the RECITAL (NCT01862926) and EvER-ILD (NCT02990286) trials will illuminate the potential role of rituximab in patients with SSc-ILD.

Nintedanib is an antifibrotic therapy that inhibits processes fundamental to the progression of lung fibrosis [40]. Nintedanib has been approved by the FDA and other regulatory bodies for reducing the rate of decline in FVC in patients with SSc-ILD, as well as for the treatment of idiopathic pulmonary fibrosis and other progressive fibrosing ILDs. The SENSCIS trial enrolled 576 patients with SSc-ILD and an extent of fibrotic ILD on HRCT (based on assessment of the whole lung) of ≥ 10%. The inclusion criteria did not require evidence of recent or ongoing progression of SSc-ILD. The rate of decline in FVC over 52 weeks was − 52.4 mL/year in the nintedanib group and − 93.3 mL/year in the placebo group, corresponding to a relative reduction of 44%. The rate of decline in FVC % predicted over 52 weeks was − 1.4%/year in the nintedanib group and − 2.6%/year in the placebo group. The side effects of nintedanib were mainly gastrointestinal and manageable for most patients through symptom management and dose adjustment [41••, 42]. Importantly, nintedanib was effective at reducing the rate of decline in FVC both when used as monotherapy and when used as add-on to a stable dose of mycophenolate [43], and had a consistent effect across subgroups of patients with SSc-ILD, including those with limited versus diffuse cutaneous SSc and ATA-negative versus ATA-positive patients [41••, 44].

Autologous hemopoietic stem cell transplantation (HSCT) is a therapeutic option for select patients with severe progressive SSc-ILD and has been recommended in treatment guidelines [45, 46]. HSCT is associated with marked improvements in disease progression and event-free survival, but also with significant short-term mortality and it should only be performed at expert centers after careful consideration of the risk:benefit for the individual patient.

Lung transplantation is available to a minority of patients with SSc-ILD who have not responded to treatment and do not have contraindications. In a retrospective study of outcomes in 90 patients with SSc at 14 European centers, survival rates 1, 3, and 5 years after transplant were 81%, 68%, and 61% [47]. Potential candidates for lung transplant should be referred to an expert center for evaluation as soon as possible.

Optimal management of patients with SSc-ILD requires a multifaceted approach, including management of symptoms, extra-pulmonary manifestations of SSc and comorbidities, and the appropriate use of non-pharmacological therapies such as pulmonary rehabilitation [48]. Supplemental oxygen should be considered for patients with severe hypoxemia [49]. Patient education and support should be regarded as an integral part of patient care [50]. Patient groups, such as those run by the Scleroderma Foundation (www.scleroderma.org), provide valuable support to communities of patients and their caregivers.

## Conclusions

ILD is a common manifestation of SSc that may become fibrosing and progressive, resulting in loss of lung function and premature death. All patients diagnosed with SSc should be screened for ILD at baseline using an HRCT scan. Regular monitoring of patients with SSc-ILD is important to evaluate progression and inform treatment decisions. The mainstay of therapy for SSc-ILD is immunosuppression. The antifibrotic drug nintedanib has demonstrated efficacy in reducing the rate of decline in FVC when used as monotherapy and as add-on to mycophenolate. A multidisciplinary approach to the assessment, monitoring, and management of SSc-ILD provides the best care for patients.
